# Serosurvey for Influenza D Virus Exposure in Cattle, United States, 2014–2015

**DOI:** 10.3201/eid2511.190253

**Published:** 2019-11

**Authors:** Simone Silveira, Shollie M. Falkenberg, Bryan S. Kaplan, Beate Crossley, Julia F. Ridpath, Fernando B. Bauermann, Charles P. Fossler, David A. Dargatz, Rohana P. Dassanayake, Amy L. Vincent, Cláudio W. Canal, John D. Neill

**Affiliations:** Universidade Federal do Rio Grande do Sul, Porto Alegre, Brazil (S. Silveira, C.W. Canal);; US Department of Agriculture, Ames, Iowa, USA (S.M. Falkenberg, B.S. Kaplan, J.F. Ridpath, R.P. Dassanayake, A.L. Vincent, J.D. Neill);; University of California, Davis, California, USA (B. Crossley);; Oklahoma State University, Stillwater, Oklahoma, USA (F.B. Bauermann);; US Department of Agriculture, Fort Collins, Colorado, USA (C.P. Fossler, D.A. Dargatz)

**Keywords:** influenza D virus, bovine, epidemiology, bovine respiratory disease, hemagglutination inhibition assay, influenza, viruses, United States, zoonoses

## Abstract

Influenza D virus has been detected predominantly in cattle from several countries. In the United States, regional and state seropositive rates for influenza D have previously been reported, but little information exists to evaluate national seroprevalence. We performed a serosurveillance study with 1,992 bovine serum samples collected across the country in 2014 and 2015. We found a high overall seropositive rate of 77.5% nationally; regional rates varied from 47.7% to 84.6%. Samples from the Upper Midwest and Mountain West regions showed the highest seropositive rates. In addition, seropositive samples were found in 41 of the 42 states from which cattle originated, demonstrating that influenza D virus circulated widely in cattle during this period. The distribution of influenza D virus in cattle from the United States highlights the need for greater understanding about pathogenesis, epidemiology, and the implications for animal health.

Influenza D virus (IDV; genus *Deltainfluenzavirus*, family *Orthomyxoviridae*) is an enveloped, single-stranded, negative sense RNA virus with 7 genome segments and 1 surface glycoprotein, the hemagglutinin-esterase fusion (HEF) protein (*1*,*2*). The first detection of IDV dates back to Oklahoma, USA, in 2011 from pigs exhibiting influenza-like disease (*3*), although retrospective seroprevalence data suggest the presence of IDV in goats in the United States before 2002 (*4*). Subsequently, IDV has been identified in low frequency in pigs in Italy (*5*,*6*) and Luxembourg (*7*). In addition, evidence suggests IDV circulates in other hosts such as small ruminants, camels, and buffalo in Togo, Kenya, and China (*8*,*9*) and small ruminants, feral swine, and equids in the United States (*4*,*10*,*11*).

Although IDV has been detected in other species, cattle appear to be the main reservoir (*1*,*12*). A variety of sample types and methods of detection have been used to determine the prevalence of IDV in different regions, in various ages, breeds, and numbers of cattle evaluated. The lack of consistency between the methods and cattle evaluated may be a contributing factor to variability in prevalence of IDV in different regions. Seroprevalence data have been reported in cattle from Luxembourg (*7*), Japan (*13*,*14*), the United States (*1*,*15*,*16*), Togo, Benin, and Morocco (*9*); the highest reported seropositive rate (80.2%) was in the United States (*16*) and Luxembourg (*7*) and the lowest (1.9%) in Benin (*9*). Serologic testing provides an indication of IDV exposure but is not a measure of active infections. IDV RNA from respiratory samples of cattle has been detected in several countries: the United States (*1*,*15*,*17*,*18*), Italy (*5*), France (*19*), Ireland (*20*), China (*8*,*21*), Japan (*22*), and Mexico (*18*). Studies from Mexico (*18*) reported the highest frequency of positive samples (29.7%) and China the lowest (0.7%) (*21*).

In both experimental and field infections with IDV, mild to moderate respiratory disease has been reported (*23*,*24*). In addition, IDV-positive samples are reported not only from cattle manifesting clinical signs associated with bovine respiratory disease but also from cattle that are asymptomatic and appear to be healthy (*20*–*22*). Experimental infection of calves demonstrated that IDV caused mild to moderate respiratory disease and that peak viral shedding occurred at 4–6 days postinfection; seroconversion was detected as early as day 6 postinfection (*12*,*23*,*24*). Whereas IDV infection by itself has been associated mainly with mild respiratory illness, IDV has also been implicated as a contributor to bovine respiratory disease complex (BRDC), which is the most costly disease affecting the US cattle industry (*17*,*18*,*23*,*25*).

Because there are no commercially available vaccines against IDV, positive serologic assays reflect natural exposure. Given the potential of IDV to contribute to BRDC, inclusion of IDV in vaccination programs has been debated. The frequency of IDV RNA–positive samples from US cattle is 4.8%–18% (*1*,*15*,*17*,*18*), and positive samples have been reported in the US cattle population since 2003 (*16*). The seropositive rate has been reported at 13.5%–80.2% (*15*,*16*); the Upper Midwest region has the highest seroprevalence. The wide variation of seroprevalence could be caused by differences in the age of the cattle evaluated or by differences across regions because of limited sample size and the focus on the Midwest and South Central regions of the country. We conducted a national serosurvey of cattle of a similar age to fully evaluate the potential role of IDV in BRDC infections and the effect of IDV on animal health and productivity.

## Materials and Methods

### Samples

We assessed 1,992 banked bovine serum samples for IDV-specific antibodies. The samples, collected between August 2014 and December 2015 as part of the US brucellosis surveillance program, were previously used to screen for ruminant pestivirus and bovine leukemia virus (BLV) exposure (*26*,*27*). We aimed to determine the seropositivity rate for IDV and retrospectively compare that rate with seropositivity rates for ruminant pestivirus and BLV from the same samples to identify regional patterns or differences in the US cattle population.

**Figure 1:**
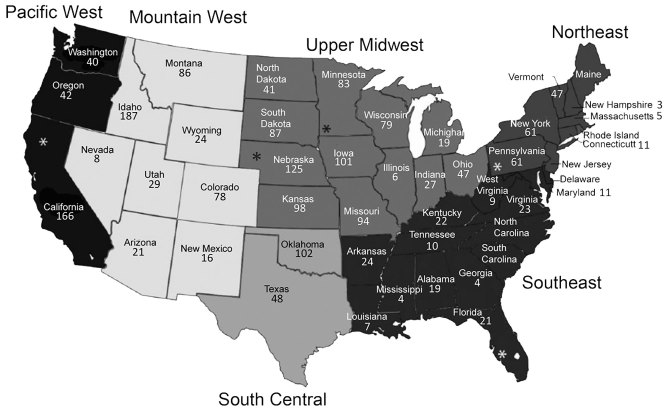
Number of samples collected from 42 states in study of influenza D virus in cattle, United States, 2014–2015. Asterisks (*) indicate states with 1 slaughter plant that contributed samples. Alaska, Hawaii, and states without numbers did not contribute samples.

The serum samples came from both male and female cattle >2 years of age, raised in 42 states, and were randomly collected from 5 slaughter plants. The states were categorized into 6 regions as previously defined (*26*): Pacific West (PW), Mountain West (MW), Upper Midwest (UMW), South Central (SC), Northeast (NE), and Southeast (SE) (Figure 1). The number of samples taken in each slaughter plant, listed by state (California, Florida, Nebraska, Pennsylvania, Minnesota), was proportional to the total annual number of cattle >2 years of age that had been processed in that plant. All samples were previously reported as negative for brucellosis.

### Virus Selection and Propagation

**Figure 2:**
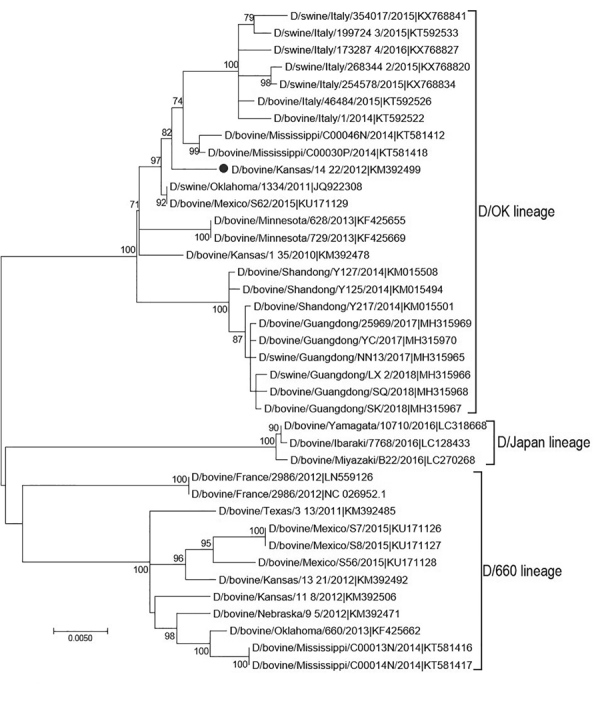
Maximum-likelihood phylogeny of the influenza D virus hemagglutinin-esterase fusion (HEF) gene constructed for study of influenza D virus in cattle, United States. Representative US strain D/bovine/Kansas/14-22/2012 (black dot), used as antigen in hemagglutination inhibition analysis, was aligned with reference strains from the Influenza Research Database (http://www.fludb.org) obtained on September 28, 2018. Bootstrap values >70% (1,000 replicates) are shown to the right of the nodes. Scale bar represents nucleotide substitutions per site.

To select the IDV strain used for the hemagglutination inhibition (HI) assay, we performed phylogenetic analysis on HEF genes with IDV strains that circulated in the United States during the same period in which the samples used for this study were collected (Figure 2). We downloaded full-length HEF gene segment sequences (n = 39) from the Influenza Research Database (http://www.fludb.org) on September 28, 2018. We aligned full-length segments using the MAFFT plug-in for Geneious version 9.1.4 (Biomatters Ltd., http://www.geneious.com) with subsequent manual correction. We constructed a maximum-likelihood tree inferred in IQ-tree (http://www.iqtree.org) using a general time-reversible model of nucleotide substitution combined with a gamma-distributed rate variation with statistical support generated through ultrafast bootstrap analysis (*28*,*29*). We chose a representative US strain, D/bovine/Kansas/14-22/12, showing a high amino acid similarity (96%–99.2%) with US strains detected during 2014–2015, and a high hemagglutination (HA) titer.

We maintained swine testicle cells (ATCC CRL-1746) used for propagation of IDV in MEM medium (Sigma Aldrich, https://www.sigmaaldrich.com), supplemented with 10% (vol/vol) heat-inactivated fetal bovine serum (PAA Laboratories, Inc., https://www.fishersci.com) and L-glutamine (ThermoFisher Scientific, https://www.thermofisher.com) antibiotic-antimycotic solution incubated at 37°C in a humid atmosphere of 5% CO_2_. We propagated the D/bovine/Kansas/14-22/12 strain, diluted 1:1,000 in swine testicle cells cultured in serum-free medium in the presence of TPCK-trypsin (0.1 µg/mL) and 5% bovine serum albumin, and incubated at 37°C for up to 4 days.

### Serology

We performed the HI assay for detection of D/bovine/Kansas/14-22/12–specific antibodies in accordance with the specifications in the World Health Organization manual on animal influenza A virus diagnosis and surveillance (*30*). We treated 1:3 serum samples with receptor-destroying enzyme (Denka Seiken UK, http://www.denka-seiken.jp) at 37°C for 18 hours, heat inactivated it at 56°C for 1 h, and diluted it 1:10 with phosphate-buffered saline. We conducted the assay in duplicate, at room temperature and in V-bottom 96-well plates, starting at 1:10 and doing 2-fold serial dilutions to reach a 1:1,280 dilution. We added the serially diluted samples to the virus (8 hemagglutination units/50 µL) for 1 h, then incubated with 0.5% turkey red blood cells for 30 min. The endpoint titer was the reciprocal of the highest dilution of serum that demonstrated partial to full inhibition of hemagglutination. We determined that a serum with an HI titer >40 was seropositive according to previous IDV serosurveillance studies (*4*,*15*). We used a negative control (PBS), as well as a positive control consisting of a rabbit polyclonal antiserum generated against D/swine/OK/1334/2011, in the HI assay (*1*). To exclude the possible presence of nonspecific antibodies, we also performed HI with serum samples from 10 colostrum-deprived calves; all showed titers of 0, which confirmed negativity.

### Statistical Analysis

We used GraphPad Prism 7 software (GraphPad Software, LLC, https://www.graphpad.com) to statistically compare seropositive rates of IDV infection by χ^2^ test and geometric mean titers (GMT) by the Kruskal-Wallis and Mann-Whitney tests. We considered p<0.05 significant.

## Results

Of the 1,992 cattle serum samples tested by HI assay for detection of IDV-specific antibodies, 1,545 (77.5%) samples were positive; the overall GMT of positive samples was 230 (titers ranged from 40 to 1,280). We identified positive serum in samples from 41 of the 42 states tested (Table). The seropositivity rate was 25%–93.8% among the states and average GMT was 80–460. However, sample size was small in some of the states with low positivity, low titer, or both, which might have caused bias in the regional distribution.

**Table:** Serosurveillance results for influenza D virus in cattle, by region and state, United States, 2014–2015*

Region and state	No. samples	Seropositive rate, %†	GMT (range)‡
Mountain West			
Idaho	187	87.2	230 (40–1,280)
Montana	86	84.9	270 (40–1,280)
Colorado	78	88.5	330 (40–1,280)
Utah	29	79.3	240 (80–1,280)
Wyoming	24	79.2	460 (80–1,280)
Arizona	21	57.1	140 (40–1,280)
New Mexico	16	93.8	210 (40–1,280)
Nevada	8	75.0	250 (80–1,280)
Total	449	84.6	260 (40–1,280)
Upper Midwest			
Nebraska	125	91.2	260 (40–1,280)
Iowa	101	92.1	270 (40–1,280)
Kansas	98	86.7	300 (40–1,280)
Missouri	94	86.2	220 (40–1,280)
South Dakota	87	90.8	300 (40–1,280)
Minnesota	83	89.2	280 (40–1,280)
Wisconsin	79	84.8	250 (40–1,280)
Ohio	47	48.9	130 (40–640)
North Dakota	41	56.1	400 (40–1,280)
Indiana	27	37.0	120 (40–640)
Michigan	19	63.2	190 (40–1,280)
Illinois	6	83.3	160 (80–320)
Total	807	84.0	260 (40–1,280)
South Central			
Oklahoma	102	79.4	230 (40–1,280)
Texas	48	75.0	170 (40–1,280)
Total	150	78.0	210 (40–1,280)
Pacific West			
California	166	77.7	190 (40–1,280)
Oregon	42	76.2	300 (40–1,280)
Washington	40	72.5	230 (40–1,280)
Total	248	76.7	210 (40–1,280)
Southeast			
Arkansas	24	83.3	180 (40–640)
Virginia	23	43.5	130 (40–640)
Kentucky	22	68.2	310 (80–1,280)
Florida	21	57.1	170 (40–1,280)
Alabama	19	68.4	140 (40–1,280)
Tennessee	10	50.0	240 (80–640)
West Virginia	9	33.3	200 (80–640)
Louisiana	7	42.9	160 (80–320)
Mississippi	4	25.0	80 (80–80)
Georgia	4	75.0	160 (80–320)
Total	143	59.5	180 (40–1,280)
Northeast			
Pennsylvania	61	50.8	120 (40–1,280)
New York	61	45.9	110 (40–1,280)
Vermont	47	51.1	110 (40–640)
Connecticut	11	0	0
Maryland	7	71.4	110 (40–1,280)
Massachusetts	5	60.0	80 (80–80)
New Hampshire	3	66.7	80 (80–80)
Total	195	47.7	110 (40–1,280)

*GMT, geometric mean titer. †Seropositive rate was calculated using those samples with hemagglutination inhibition titer ≥40.  ‡GMT was calculated using those samples with HI titer ≥40. Lowest and highest titers were measured from those samples with HIT titer >40.

We categorized the results by geographic region to compare the differences of seropositive rate and GMT. The seropositive rate range was 47.7%–84.6% (p<0.05) and GMT 110–260 (p<0.05) among the regions (Table). Mountain West region had the highest seropositive rate (84.6%) and GMT (260); Northeast region had the lowest seropositive rate (47.7%) and GMT (110).

## Discussion

Although IDV was described in pigs earlier than in cattle in the United States, subsequent reports of retrospective samples suggested that cattle are the natural reservoir (*1*,*12*). Because seroprevalence surveillance in US cattle had been conducted only at state or regional levels, we undertook a nationwide serologic survey to detect IDV antibodies in cattle. Our results clearly demonstrate that IDV circulated with high frequency in cattle in all regions of the United States during 2014–2015.

We observed regional variation in seropositive rate and GMT, although all regions had relatively high frequency. Overall, the Upper Midwest and Mountain West regions showed the highest seropositive rates and the highest antibody titers, and also encompassed the states with the highest GMT. A similar result was obtained in a pestivirus serologic study performed with the same serum samples; here too, the Mountain West region showed the highest number of antibody-positive animals and higher titers (*26*). Although it is not possible to establish the cause, both pestivirus and IDV serology follow a similar trend. Potential causes include herd size, which can exceed 1,000 animals in these areas, and the potential for livestock and wildlife species to commingle and facilitate virus transmission. Evidence indicates IDV can infect nonbovine hosts, such as sheep, goats, pigs, and equids, in the United States (*4*,*10*,*31*). However, the full range of susceptible hosts for IDV is unknown, and interspecies transmission has not been demonstrated among the known hosts. 

Seroprevalence of IDV in small ruminants was reported in samples collected from the Mountain West and Upper Midwest regions, whereas samples from other regions were negative (*4*). Moreover, in the Upper Midwest region, a high percentage of small ruminants with high titers was described, and the farms where they were located were in close proximity to cattle farms (*4*). This issue needs to be explored further to understand the importance of IDV as a threat for animal health and whether this is an underlying factor for the increased seroprevalence of viral pathogens in regions that have greater potential for interspecies transmission.

In general, we observed lower titers and a lower percentage of positive animals in the Northeast and Southeast regions. These results are similar to those reported from the pestivirus serosurvey that also found these 2 regions to have the lowest titers and lowest number of cattle seropositive for BVDV (*26*). On the other hand, in the BLV serosurvey, the Northeast had the highest seropositive rate for BLV and the Mountain West the lowest seropositive rate (*27*). Although seroprevalence differences existed between BLV and the other viruses evaluated (pestivirus and IDV), these differences could be caused by limited number of samples collected in these regions, differences in the epidemiology of these viruses, or differences in herd management practices across the regions. Previous data of IDV exposure in cattle of different ages in Mississippi (Southeast region) reported a high seroprevalence in cattle >1 year of age (*15*). Discrepancies between the current study and the previous reports could be explained by the number of samples evaluated in each of these studies; only 4 samples originated from Mississippi in our study, whereas >500 cattle were sampled in a previous study (*15*). Although our study encompassed the entire United States, the limited number of samples from several states, and subsequently the regions they represent, may have caused underestimation or overestimation of the seropositive rate of IDV. Despite the limitations of our study, data indicate that IDV is widespread at rates similar to the regional or state data previously reported (*15*,*16*).

Our findings, combined with those from previous serosurveillance studies (*15*,*16*), confirm a high nationwide seroprevalence of IDV in US cattle populations. Because of the potential association of IDV with BRDC (*17*,*18*,*23*,*31*) and the dearth of vaccines to prevent IDV infection (*12*,*32*), concerns have been raised regarding the negative effect of IDV on animal health. A possible explanation for the high seropositive rate is that IDV is common in the respiratory tract of cattle; times of stress, immune attack, or environmental changes that affect the respiratory tract can increase viral shedding but might not cause disease. Unpublished diagnostic data from our laboratory show that IDV is detected more frequently in samples that are also positive for other respiratory pathogens than in those positive for IDV alone. This finding indicates that IDV can either predispose the respiratory tract or act as an opportunistic pathogen in concert with other pathogens to cause BRD. Further research, including co-infection studies, is needed to elucidate the full range of susceptible hosts and the dynamics of interspecies transmission to understand the contribution of IDV to BRDC. In summary, our serosurveillance study of bovine serum samples from 2014–2015 showed a high seropositivity rate for IDV in the United States; 41 of the 42 states from which cattle originated had seropositive animals. No IDV vaccine exists. IDV infection has also been implicated in BRDC, the most costly disease affecting the US cattle industry. Therefore, our findings may indicate an ongoing risk to animal health. 
